# Predicting CBT modality, treatment participation, and reliable improvements for individuals with anxiety and depression in a specialized mental health centre: a retrospective population-based cohort study

**DOI:** 10.1186/s12888-024-05817-w

**Published:** 2024-05-23

**Authors:** Afsaneh Roshanghalb, Bojay Hansen, David Rudoler, Michael W Best

**Affiliations:** 1 Faculty of Health Sciences, The University of Ontario Institute of Technology, Oshawa, Ontario Canada; 2 Ontario Shores Centre for Mental Health Sciences, Whitby, Ontario Canada; 3 Departments of Psychology and Psychological Clinical Science, University of Toronto, Scarborough, Ontario Canada

**Keywords:** Cognitive behaviour therapy, Modality, Deprivation index, Reliable and clinically significant improvement (RCSI), Administrative data, Anxiety, Depression

## Abstract

**Background:**

Cognitive Behaviour Therapy (CBT) is one of the most successful therapeutic approaches for treating anxiety and depression. Clinical trials show that for some clients, internet-based CBT (eCBT) is as effective as other CBT delivery modes. However, the fidelity of these effects may be weakened in real-world settings where clients and providers have the freedom to choose a CBT delivery mode and switch treatments at any time. The purpose of this study is to measure the CBT attendance rate and identify client-level characteristics associated with delivery mode selection and having reliable and clinically significant improvement (RCSI) of treatment in each delivery mode in a real-world CBT outpatient program.

**Methods:**

This is a retrospective cohort analysis of electronic medical records collected between May 1, 2019, and March 31, 2022, at Ontario Shores Centre for Mental Health Sciences. Regression models were used to investigate the impact of individual client characteristics on participation and achieving RCSI of different CBT delivery modes.

**Results:**

Our data show a high attendance rate for two and more CBT sessions across all modalities (98% of electronic, 94% of group, 100% of individual, and 99% of mixed CBT). Individuals were more likely to enter mixed and group CBT modality if they were younger, reported being employed, and reported higher depression severity at the baseline. Among the four modalities of CBT delivery, group CBT clients were least likely to have RCSI. Of those who started sessions, clients were significantly more likely to experience RCSI on the Patient Health Questionnaire (PHQ)-9 and the Generalized Anxiety Disorder (GAD)-7 if they were employed, reported more severe symptoms at baseline, and were living in the most deprived neighborhoods.

**Conclusions:**

This study will contribute to the body of knowledge about the implementation and treatment planning of different CBT delivery modes in real-world settings. With the changing clinical environment, it is possible to advocate for the adoption of the eCBT intervention to improve therapy practices and achieve better treatment success. The findings can help guide future CBT program planning based on client socio-demographic characteristics, allowing the optimal therapy type to be targeted to the right client at the right time.

**Supplementary Information:**

The online version contains supplementary material available at 10.1186/s12888-024-05817-w.

## Introduction and background

Anxiety and depression are two common mental health conditions that have been successfully treated using cognitive behavioural therapy (CBT), a widely used therapeutic method. The research literature has established the effectiveness of several CBT delivery methods, including individual, group, and internet-based therapy with coaching [1–4]. There is evidence that CBT is an effective treatment for variety of mental disorders such as generalized anxiety disorder [5], depression [6], panic disorder [7]. There is also strong evidence that suggests internet-based cognitive behavioural therapy (eCBT) is equally as effective as individual treatment for depression [3, 8] and anxiety [9]. The Internet is a good way to spread preventive measures because the units of delivery are cheaper and easy to use [10]. eCBT has become an important modality during the COVID-19 pandemic as access to individual treatment is limited. Understanding the challenges in delivering different CBT modalities will be essential for improving depression and anxiety treatments and implementation of stratified care in specialized mental health centres.

The use of CBT therapies in real-world settings requires further study because a large portion of this evidence relies on controlled experimental settings. The main drawback of these clinical trials is that the implementation of the interventions typically involves a high degree of fidelity to recommended practises and client adherence. This level of fidelity and adherence is likely to be lower in real-world contexts and less is known on what client characteristics are associated with entering therapy modalities and which client variables are connected with completing treatment [11–13].

Past empirical studies assessed CBT initiation and dropout rates using variety of client related factors, yet they have limited agreement on the strength of the evidence for these factors. As discussed by El Alaoui et al. [5], this may be the result of a variety of factors, including variations in dropout definition, sample size and features, and statistical techniques for analysing the prognostic significance of baseline variables. This study assesses the relationship between client characteristics on participation in and completion of treatment in different CBT modalities (eCBT, group, individual, mixed) to identify factors that may influence client success in different CBT modalities. The aim is to identify opportunities for further optimization of treatment assignment.

## Materials and methods

### Study setting

This study used retrospective cohort design, and administrative data were taken from the electronic medical records (EMRs) at Ontario Shores Centre for Mental Health Sciences in Ontario, Canada. Ontario Shores is a specialized mental health hospital in Ontario, Canada that serves a regional population of 2.8 million people. The hospital has 18 care units, 346 inpatient beds, and has over 94,000 outpatient visits annually. The proposed research will support the evaluation and continuous improvement of a multi-modal CBT program at Ontario Shores. Interventions in this program are provided by more than 30 clinicians (social workers, nurses, occupational therapists, and psychologists) across 30 sites.

Ontario Shores Anxiety and Mood Program (AMD) offers individual, group, eCBT (or internet-based CBT with coaching) and mixed modality that is a combination of two or three of these modalities offered to the patient. Individual therapy comprises 60-minute face-to-face structured psychotherapy sessions with a dedicated therapist. Group therapy involves in-person structured psychotherapy led by a single therapist and attended by two or more clients. To address mild to moderate depression and anxiety, Ontario Shores introduced eCBT on May 1, 2019. This modality incorporates coaching sessions, self-help learning modules, informational resources, and assigned homework to reinforce key concepts and impart mental health skills. Clients participating in eCBT, akin to other modalities, have the option to complete assessments throughout the program to monitor progress and enhancements. Aligned with the BC-CBT framework, this therapeutic approach seamlessly integrates in-person sessions with digital and online components for a comprehensive and flexible treatment experience.

Modality selection is determined by a client’s diagnosis, symptoms, and personal preferences. Based on the shared decision-making principals, mental healthcare providers will discuss the different potential treatment options with the client and come to an agreement on the best modality.

### Data

Clinical staff members gathered and entered outpatient data inputted into the EMRs (a system called Meditech) at each client interaction. The outpatient records were collected for clients with an initial pre-session evaluation and at least one session of individual, group, or eCBT offered by Ontario Shores. The study utilised data acquired between May 1, 2019, and March 31, 2021, with a one-year follow-up through March 31, 2022, to assess the treatment completion of all clients. We looked back two years to ensure that the first client encounter had been included. For all clients, the first encounter date is the date of their initial pre-session evaluation prior to starting CBT treatment. The EMR data included demographic information from individuals who received eCBT, group CBT, individual CBT, or a combination of these treatments, as well as information from Integrated Community Access Program (ICAP) clinics that provide psychotherapy and medication for individuals with anxiety and mood disorders. At the time of data collection, our exclusion criteria were limited to 31 records pertaining to 5 distinct individuals for whom a confidential flag had been applied. Furthermore, we did not have access to data on services provided by the Ontario Structured Psychotherapy Program, which is a parallel dissemination of CBT occurring in the province during this period with unique treatment and data acquisition protocols.

### Outcome measures

Outcome measures included CBT modality selection (i.e., individual, group, eCBT, mixed) and reliable and clinically significant improvement (RCSI) in symptoms of anxiety and depression from baseline to end of follow-up. In practice, healthcare providers will choose the best available modality and advise patients, accordingly, taking the client’s diagnosis and symptoms into consideration. Symptoms of anxiety were measured using the 7-item Generalized Anxiety Disorder (GAD-7) scale [14]. Depressive symptoms were measured using the 9-Question Patient Health Questionnaire (PHQ-9) [15]. Both measures have been validated previously and are commonly employed in clinical settings [16, 17]. The RCSI has been calculated by data and analytics professionals to support treatment planning and routine outcome monitoring inside the Ontario Shores Hospital. In the context of the CBT program, RCSI was defined as a shift in PHQ-9 of > = 6 points from baseline to final scores at the treatment’s conclusion, with an initial PHQ-9 score of > = 10. Similarly, GAD-7 changes > = 5 points from baseline to final scores at the end of treatment, where the first GAD-7 score was > = 8, signifying successful treatment [18, 19].

### Data preparation

Our primary sample included 16,510 sessions for 1,098 unique client records who received CBT sessions in the Ontario Shores Mood and Anxiety clinics during the study period. In total, 6,864 electronic CBT, 3,996 group CBT, and 8,808 individual CBT sessions have been provided. All our sample started treatment and 97% completed two or more sessions.

Data management steps included excluding part of independent variables with more than 20% missing information. The decision to exclude variables with high percentages of missing information was primarily driven by the acknowledgment of poor data quality in out-patient databases. Imputing or attempting to fill in missing values for these variables might lead to biased or unreliable results, and therefore, it was deemed more appropriate to remove them from the list of variables considered for analysis. The variables retained in the analysis have undergone this careful data management process to enhance the overall validity of our research findings. We excluded sociodemographic characteristics with high percentage of missing information, such as education (98% missing) and racial identity (29% missing) as well as clinical variables such as primary diagnoses (28% missing) and secondary diagnoses (40% missing). For variables with low levels of missing data, including living arrangement (1%), employment status (6%), Canadian Index of Multiple Deprivation (CIMD) (5%), baseline GAD-7 (6%), and baseline PHQ-9 (less than 1%), we opted for Multiple Imputation (MI). This approach addresses missing values statistically, enhancing the completeness of the dataset and ensuring robustness in subsequent analyses. The MI process involved generating substitute values tailored to the type of each independent variable. In the end, no observations were dropped due to missing data.

Specifically, Bayesian linear regression was applied for continuous variables such as baseline client outcomes, logistic regression for dichotomous variables like living and employment status, and Polytomous logistic regression for categorical variables such as neighborhood deprivation. This approach ensured a comprehensive imputation strategy considering the diverse nature of the dataset’s variables. We utilized RStudio’s “mice” function within the “mice” package to implement MI.

### Statistical analysis

Descriptive statistics were performed to compile client variables relevant to each CBT modality that were obtained from the client’s screening (i.e., age, sex, employment, and living status).

We estimated a multinomial logit regression for *n* = 1,098 clients to determine what client characteristics are related to entering each therapy modalities (Individual, group, eCBT, and mixed). We chose eCBT as the basis category due to its higher frequency. Using “multinom” function from “nnet” RStudio package, we modelled modality choice as a function of client age (adults between the ages of 17–70), sex at birth (male or female), living situation (alone or in a household), employment situation (employed or unemployed), Canadian Index of Multiple Deprivation (in five quantiles), baseline GAD-7, and baseline PHQ-9. The CIMD is a composite index that assesses various dimensions of deprivation and socioeconomic well-being across different geographic areas in Canada. Developed by Statistics Canada, the CIMD integrates multiple indicators related to income, education, employment, housing, and living conditions [20] to provide a comprehensive measure of deprivation. The index assigns quantitative scores to geographic areas based on the levels of deprivation observed. Higher scores indicate higher levels of deprivation, while lower scores suggest better socioeconomic conditions.

To answer our second research question on the predictors of having RCSI, we estimated a logistic regression model using “glm” function for post-treatment RSCI outcome in the PHQ-9 and GAD-7 measures. The outcomes were modelled as a function of CBT modality (Individual, group, eCBT, and mixed), age (adults between the ages of 17 and 70), sex at birth (male or female), living situation (alone or with others), employment situation (employed or unemployed), CIMD deprivation Index (in five quantiles), and baseline GAD-7 and PHQ-9 scores.

## Results

### Baseline data

Table 1 shows descriptive statistics for clients receiving electronic (n = 563), group (n = 143), individual (n = 256), and mixed CBT (n = 136). The findings are based on the completed (n = 711) imputed data (n = 387) for all 1,098 clients receiving CBT at Ontario Shores between May 1, 2019, and March 31, 2022. The analysis of baseline PHQ-9 and GAD-7 scores reveals a consistent pattern across most Cognitive Behavioral Therapy (CBT) modalities. However, an exception is observed in the case of eCBT. In the eCBT group, the average baseline GAD-7 score was 12 (SD = 5.1), compared to an average of 14 in the other groups (F-statistic = 9.209, p = 5.55e-06. This places the average score within the category of ‘Moderate anxiety’ (10–14 range) according to the GAD-7 scoring guidelines [14]. In the eCBT group, the average baseline PHQ-9 score was 13 (SD = 5.8), compared to an average of 16 to 18 in the other groups (F-statistic = 25.45, *p* = 1.21e-15) This information suggests that individuals participating in or being assigned to eCBT may, on average, exhibit more moderate levels of depression compared to counterparts in other CBT modalities.

**Table 1:** Baseline data on selected demographics of patients who received CBT at Ontario Shores between 2019 and 2022

Characteristics	Electronic CBT		Group CBT		Individual CBT		Mix^1^ CBT		Overall Sample	
	*n*= 563		*n*=143		*n*=256		*n*=136		*n*=1,098	
Female	388	69%	99	69%	166	65%	93	68%	746	68%
Living with Other People	493	88%	124	87%	230	90%	119	88%	966	88%
Employed	313	56%	59	41%	86	34%	47	34%	505	46%
Completed two sessions and more	550	98%	135	94%	256	100%	129-135	95-99%	1076	97%
Completed six sessions and more	450	80%	108	76%	246	96%	124	91%	928	85%
RCSI^2^	299	53%	79	55%	143	56%	69	51%	590	54%
CIMD^3^ Neighborhood Deprivation										
* Quintile 1*	95	17%	21	15%	49	19%	13	10%	178	16%
* Quintile 2*	176	31%	35	24%	69	27%	39	29%	319	29%
* Quintile 3*	111	20%	35	24%	58	23%	39	29%	243	22%
* Quintile 4*	95	17%	20	14%	42	16%	14	10%	171	16%
* Quintile 5*	86	15%	32	22%	38	15%	31	23%	187	17%
Age at admission, Mean (SD)	34 (12.62)		37 (14.01)		32 (13.29)		37 (13.08)		34 (13.12)	
Number of attended appointments, Mean (SD)	9 (5.30)		14 (10.83)		25 (18.30)		21 (12.69)		15 (13.04)	
GAD-7 Baseline Score, Mean (SD)	12 (5.11)		14 (4.82)		14 (5.20)		14 (4.66)		14 (5.14)	
PHQ-9 Baseline Score, Mean (SD)	13 (5.76)		16 (5.55)		18 (6.40)		17 (5.29)		17 (6.06)	

^1^Two or three different CBT techniques were used as part of the therapy plan

^2^Post-treatment Reliable and Clinically Significant Improvement in the PHQ-9 and GAD-7 measures

^3^Canadian Index of Multiple Deprivation

Among all modalities, clients undergoing individual CBT exhibited the highest attendance rate averaging 25 sessions. Clients on average were young females living with others in a moderate socioeconomically deprived neighborhood (Q2 and Q3). They were mostly unemployed, and they had moderate anxiety (10–14 GAD-7 scores risk level) and moderately severe depression (15–19 PHQ-9 scores risk level) on average, with the eCBT cohort scoring the lowest symptom severity. At the end of treatment, more than half of the clients have attained RCSI (56% individual CBT, followed by 55% group, 53% electronic, and 51% mixed CBT).

### Multinomial regression findings

To answer our first research question on the characteristics associated with entering each CBT modality, we developed a multinomial logistic regression with modality type as the dependent variable. Table 2 displays coefficient results of each CBT modality in comparison to our baseline modality which is eCBT. Employment was associated with lower probability of receiving individual CBT (OR = 0.49, 95% CI [0.35–0.68]), mixed CBT (OR = 0.5, 95% CI [0.33–0.75]), and group CBT (OR = 0.64, 95% CI [0.44–0.95]). Older age was a predictor of entering into the mixed modality (OR = 1.02, 95% CI [1.01–1.04]) and group CBT (OR = 1.02, 95% CI [1.00-1.03]) versus eCBT. Although the results of CIMD neighborhood deprivation index were inconsistent, living in the fifth quantile (Q5), which identifies the most deprived neighborhood [20], was associated with participation in mixed CBT (OR = 2.24, 95% CI [1.08–4.63]). Compared to eCBT, the severity of the client’s depressive symptoms at baseline was a positive predictor of receiving individual (OR = 1.15, 95% CI [1.11–1.20]), mixed (OR = 1.08, 95% CI [1.03–1.13]), and group CBT (OR = 1.06, 95% CI [1.02–1.11]).

**Table 2:** Results of modeling multinomial regression analysis of entering treatment for each modality versus eCBT

Variables	Group CBT Vs. eCBT, *N*=143						Individual CBT Vs. eCBT, *N*=256						Mix CBT Vs. eCBT, *N*=136					
	β	SE	OR	95% CI for OR			β	SE	OR	95% CI for OR			β	SE	OR	95% CI for OR		
				Low	High					Low	High					Low	High	
Age	0.02	0.01	1.018	1.004	1.033	*^1^	-0.01	0.01	0.991	0.979	1.003		0.02	0.01	1.021	1.007	1.036	**
Sex																		
Female (ref)																		
Male	0.02	0.21	1.018	0.676	1.533		0.24	0.17	1.269	0.905	1.779		0.07	0.21	1.069	0.703	1.626	
Living Status																		
Living Alone (ref)																		
Living with other people	-0.03	0.29	0.975	0.555	1.715		0.08	0.26	1.084	0.650	1.808		0.05	0.30	1.056	0.583	1.914	
Employment Status																		
Unemployed (ref)																		
Employed	-0.44	0.20	0.645	0.439	0.948	*	-0.71	0.17	0.493	0.355	0.685	***	-0.69	0.21	0.502	0.335	0.752	**
CIMD Neighborhood Deprivation																		
Quintile 1 (ref)																		
Quintile 2	-0.23	0.31	0.798	0.434	1.467		-0.37	0.24	0.689	0.427	1.112		0.31	0.35	1.368	0.686	2.728	
Quintile 3	0.25	0.32	1.284	0.692	2.381		-0.10	0.26	0.904	0.546	1.496		0.80	0.36	2.230	1.109	4.487	*
Quintile 4	-0.22	0.35	0.799	0.400	1.594		-0.32	0.28	0.728	0.423	1.253		-0.17	0.42	0.846	0.371	1.929	
Quintile 5	0.40	0.33	1.491	0.788	2.823		-0.38	0.28	0.687	0.394	1.195		0.80	0.37	2.236	1.080	4.630	*
GAD-7 at Baseline	0.02	0.02	1.017	0.969	1.068		-0.02	0.02	0.981	0.942	1.021		0.00	0.03	1.001	0.952	1.051	
PHQ-9 at Baseline	0.06	0.02	1.065	1.021	1.110	**	0.14	0.02	1.155	1.114	1.199	***	0.08	0.02	1.083	1.037	1.131	***

^1^Legend for p-value codes: 0.001 ‘***’ 0.01 ‘**’ 0.05 ‘*’ 0.1 ‘.’

### Logistic regression findings on reliable improvement outcome

Table 3 shows the results of a logistic regression analysis on the post-treatment RCSI outcome. From our sets of demographic variables employment (OR = 1.35, 95% CI [1.03–1.77]) was significant in predicting treatment success in terms of having RCSI. As expected, both baseline PHQ-9 (OR = 1.11, 95% CI [1.07–1.15]) and GAD-7 (OR = 1.07, 95% CI [1.04–1.11]) are significantly associated with RCSI. Regarding the modality type, participating in individual (OR = 0.69, 95% CI [0.49–0.98]) and mixed CBT (OR = 0.67, 95% CI [0.44–1.01]) modalities decreased the probabilities of having RCSI relative to eCBT. Despite the inconsistency of the CIMD quantile results, living in the most deprived neighborhood (Q5) (OR = 0.60, 95% CI [0.38–0.94]) decreased the likelihood of having post-treatment RCSI.

**Table 3:** Logistic regression results of the post-treatment RCSI

Variables	β	SE	Odds Ratio (OR)	95% Confidence Interval (CI) for OR		
Age	-0.01	0.01	0.995	0.985	1.005	
Sex						
* Female (ref)*						
Male	-0.02	0.14	0.977	0.741	1.290	
Living Status						
* Living Alone (ref)*						
Living with other people	0.22	0.20	1.252	0.843	1.860	
Employment Status						
* Unemployed (ref)*						
Employed	0.30	0.14	1.347	1.031	1.764	*^1^
CBT Modality type						
* eCBT (ref)*						
Group	-0.18	0.21	0.837	0.558	1.257	
Individual	-0.36	0.18	0.694	0.491	0.980	*
Mixed	-0.40	0.21	0.667	0.441	1.007	.
GAD-7 at Baseline	0.10	0.02	1.109	1.074	1.147	***
PHQ-9 at Baseline	0.07	0.01	1.074	1.044	1.106	***
CIMD Neighborhood Deprivation						
* Quantile 1 (ref)*						
Quantile 2	-0.40	0.20	0.670	0.448	0.999	.
Quantile 3	-0.28	0.22	0.757	0.495	1.156	
Quantile 4	-0.31	0.24	0.732	0.461	1.160	
Quantile 5	-0.51	0.23	0.600	0.382	0.939	*

^1^Legend for p-value codes: 0.001 ‘***’ 0.01 ‘**’ 0.05 ‘*’ 0.1 ‘.’

## Discussion

The purpose of this study is to investigate the relationship between client-level characteristics and participation in different CBT modalities. Furthermore, we examine the predictors of CBT post-treatment RCSI using a retrospective cohort design. In line with previous studies such as Luo et al. (2020) [22], our findings revealed that electronic CBT, along with the other three modes of CBT delivery, had a high attendance rate (showing low early dropout rates), with approximately 80% of clients completing at least six sessions or more. Post-treatment RCSI of PHQ-9 and GAD-7 was present in about 50% of the CBT attendees. Our adjusted findings show lower relative odds of RCSI in more traditional therapy options (individual, group) compared to eCBT, and absolute (unadjusted) differences in probability of RCSI was similar across modalities (from 53% in eCBT to 56% in individual). A notable initial finding reveals a significant variation in the average number of sessions across different modalities, spanning from 9 sessions for eCBT to 25 sessions for individual CBT. It is important to note that while eCBT can be effective for many individuals, it may not be suitable for everyone. Certain conditions or personal circumstances may require the support and guidance of a therapist in a traditional therapy setting. The consultation with a mental health professional can determine the most appropriate form of therapy for client’s specific needs.

There is a large body of literature on CBT treatment initiation or dropout prediction [23–26] that focuses on dropout rates based on demographic and clinical variables. This study adds to this literature by investigating the individual-level predictors of treatment across a variety of CBT delivery modes to inform program planning. While our findings are descriptive, they suggest that clients with higher baseline PHQ-9 scores (indicating more severe symptoms) are referred to more intensive CBT (e.g., individual, group and mixed). Despite the importance of baseline GAD-7 scores, they were not independently important and associated with treatment modality when adjusting for PHQ-9. There are few differences in other client characteristics across the four treatment modalities. Though, one finding that merits further investigation is that being employed increased the likelihood of receiving eCBT for depression and anxiety disorders. Older clients were also more likely to receive mixed and group CBT therapy.

Our study on the Ontario Shores CBT program highlights the significance of considering clients’ socio-demographic variables, such as employment status, when assessing the success rates of CBT treatment across different modalities. Incorporating predictive algorithms based on sociodemographic factors can offer valuable insights for making informed decisions regarding the appropriate type of psychotherapy treatment for clients.

Regarding modality type, our findings indicate that clients with more severe symptoms are referred into higher intensity CBT and are less likely to respond to treatment. This suggest that there is opportunity to look to other treatments in cases of non-response. In this population, eCBT was at least as effective as more conventional therapies. While this is descriptive, it may imply that there are additional opportunities to optimize care by referring more clients to eCBT given it is less resource intensive.

## Study limitation

In our study, certain limitations must be addressed. Firstly, we encountered data quality issues for this study and were unable to work with a broader range of important patient and treatment related variables; consequently, four independent variables were eliminated from our analysis. Secondly, the exclusion of individuals with confidential EMRs was necessary to safeguard requested privacy. Additionally, at the time of data pooling, the unique treatment and data acquisition protocols of OSP led to its exclusion.

The choice of the methodology from authors was based on the nature of our data. We did not consider longitudinal models as all patient characteristics were collected at admission to the CBT program, and most were time-invariant. We collected GAD-7 and PHQ-9 scores longitudinally to calculate the RCSI outcome. However, many clients received only two measurements over their length-of-stay – typically at admission at end-of-treatment. Thus, we had limited ability to capture and analyze changes or over time.

One additional question regarding the data was whether the COVID-19 pandemic had an impact on the outcomes. Using a sample of 116 people who participated in each of the 4 modalities, we performed a sensitivity analysis to count for any demographic variations in comparison to the larger sample to verify this matter (details in the appendix section). Our research cannot establish causality; its findings are primarily descriptive. Given this is an observational study, our results should not be interpreted causally due to unobserved confounding.

This study is limited to a single hospital in Ontario, Canada, and the results may not be applicable to other settings. Ontario Shores offers a variety of CBT treatment alternatives for patients diagnosed with depression and anxiety, including eCBT from May 2019 that made the treatment more accessible and less expensive since the onset of COVID-19. Based on our original findings, eCBT appears to be effective for a specific population, and there may be a case for expanding its use. It is important to note that this is a real-world intervention implemented in an uncontrolled clinical setting, the collection of assessment data was not always consistent. Thus, we used the last available scores for each client to assess RCSI prior to patients leaving the program. The one-year follow-up allowed us to follow all clients from admission to the end of their treatment as clients had variable treatment duration and they may have achieved RCSI prior to their final assessment and that the goal is not to assess long term sustained remission of the condition, but only the state of symptoms at the end of treatment.

## Conclusions

The results indicate that there is a room for optimization of the selection process regards to different CBT types for clients admitted with depression and anxiety disorders. Baseline symptoms of depression is an important predictor of treatment success and modality selection. However, those who receive eCBT are more likely to see reliable improvement in their symptoms. This might suggest opportunity to use baseline client characteristics as a predictor of optimal treatment selection. Further research is needed to assess the generalizability of the results and incorporate a broader range of clinical and individual factors, such as client medications, diagnosis, and preferences, into models that shape practitioners’ program recommendations and predict the outcomes of the entrance and modality options.

### Electronic supplementary material

Below is the link to the electronic supplementary material.

Supplementary Material 1

Supplementary Material 2

Supplementary Material 3

Supplementary Material 4

## Data Availability

This study utilised electronic health data and client chart reviews acquired at each client encounter at Ontario Shores Hospital. Officers at Ontario Shores have preserved the integrity and confidentiality of the dataset. The data that support the findings of this study are available from Ontario Shores Centre for Mental Health Sciences but restrictions apply to the availability of these data, which were used under license for the current study, and so are not publicly available.

## Abbreviations

CBT: Cognitive Behaviour Therapy
EMRs: Electronic Medical Records
ICAP: Integrated Community Access Program
RCSI: Post-treatment Reliable and Clinically Significant Improvement in the PHQ-9 and GAD-7 measures
LOS: length of stay
MI: Multiple Imputation
CIMD: Canadian Index of Multiple Deprivation
eCBT: Internet-based Cognitive Behaviour Therapy
UP: Unified protocol
PHQ-9: 9 items Patient Health Questionnaire
GAD-7: 7 items General Anxiety Disorder questionnaire
