# Supplementary material for: Predicting CBT modality, treatment participation, and reliable improvements for individuals with anxiety and depression in a specialized mental health centre: a retrospective population-based cohort study
Source: BMC Psychiatry. 2024 May 23;24:390. doi: 10.1186/s12888-024-05817-w (PMC11112857; doi:10.1186/s12888-024-05817-w)
Supplement: Supplementary file 1 — Supplementary Material 1 [file 12888_2024_5817_MOESM1_ESM.docx]

Table S1. Client characteristics of sample of 155 clients who attended CBT before COVID-19 pandemic.

| **Characteristics** | **Electronic CBT** | | **Group CBT** | | **Individual CBT** | | **Mix* CBT** | | **Overall Sample** | |
| --- | --- | --- | --- | --- | --- | --- | --- | --- | --- | --- |
|  | **n= 39** | | **n= 39** | | **n= 45** | | **n= 32** | | **N = 155** | |
| Female | 27 | 69% | 29 | 74% | 27 | 60% | 26 | 81% | 109 | 70% |
| Living with Other People | 32 | 82% | 30 | 77% | 39 | 87% | 29 | 91% | 130 | 84% |
| Employed | 22 | 56% | 18 | 46% | 11 | 24% | 15 | 47% | 66 | 43% |
| Completed six sessions and more | 37 | 95% | 19 | 49% | 45 | 100% | 21 | 66% | 122 | 79% |
| RCSI | 25 | 64% | 24 | 62% | 31 | 69% | 17 | 53% | 97 | 63% |
| CIMD Neighborhood Deprivation |  |  |  |  |  |  |  |  |  |  |
| Quantile 1 | S^[[1]](#footnote-1)^ | S | S | S | 13 | 29% | S | S | 25 | 16% |
| Quantile 2 | 16 | 41% | 11 | 28% | 10 | 22% | 12 | 38% | 49 | 32% |
| Quantile 3 | 6-9 | 15%-23% | 6-9 | 15%-23% | 8 | 18% | 9 | 28% | 32 | 21% |
| Quantile 4 | S | S | S | S | S | S | S | S | 17 | 11% |
| Quantile 5 | 6-9 | 15%-23% | 11 | 28% | 9 | 20% | S | S | 32 | 21% |
| Age at admission, Mean (SD) | 31 (13.22) | | 41 (13.83) | | 30 (13.40) | | 37 (14.28) | | 34 (14.21) | |
| GAD-7 Baseline Score, Mean (SD) | 14 (5.88) | | 15 (4.66) | | 15 (4.40) | | 13 (5.20) | | 13 (5.57) | |
| PHQ-9 Baseline Score, Mean (SD) | 15 (6.54) | | 17 (5.81) | | 20 (4.71) | | 17 (5.92) | | 17 (5.6) | |

1. s = suppressed due to small cell size. [↑](#footnote-ref-1)
