# Supplementary material for: Predicting CBT modality, treatment participation, and reliable improvements for individuals with anxiety and depression in a specialized mental health centre: a retrospective population-based cohort study
Source: BMC Psychiatry. 2024 May 23;24:390. doi: 10.1186/s12888-024-05817-w (PMC11112857; doi:10.1186/s12888-024-05817-w)
Supplement: Supplementary file 2 — Supplementary Material 2 [file 12888_2024_5817_MOESM2_ESM.docx]

Table S2. Multinomial results of a sensitivity analysis for modelling the entrance in each treatment modality vs. eCBT.

|  | **Group CBT Vs. eCBT, n = 39** | | | | | | **Individual CBT Vs. eCBT, n = 45** | | | | | | **Mix CBT Vs. eCBT, n = 32** | | | | | |
| --- | --- | --- | --- | --- | --- | --- | --- | --- | --- | --- | --- | --- | --- | --- | --- | --- | --- | --- |
| **Variables** | β | SE | OR | 95% CI for OR | |  | β | SE | OR | 95% CI for OR | |  | β | SE | OR | 95% CI for OR | |  |
|  |  |  |  | Low | High |  |  |  |  | Low | High |  |  |  |  | Low | High |  |
| Age | 0.06 | 0.02 | 1.057 | 1.017 | 1.099 | ** | 0.00 | 0.02 | 1.000 | 0.960 | 1.042 |  | 0.04 | 0.02 | 1.037 | 0.996 | 1.080 |  |
| Sex |  |  |  |  |  |  |  |  |  |  |  |  |  |  |  |  |  |  |
| Female (ref) |  |  |  |  |  |  |  |  |  |  |  |  |  |  |  |  |  |  |
| Male | -0.25 | 0.56 | 0.776 | 0.257 | 2.345 |  | 0.32 | 0.55 | 1.377 | 0.469 | 4.039 |  | -0.66 | 0.63 | 0.518 | 0.151 | 1.776 |  |
| Living Status |  |  |  |  |  |  |  |  |  |  |  |  |  |  |  |  |  |  |
| Living Alone (ref) |  |  |  |  |  |  |  |  |  |  |  |  |  |  |  |  |  |  |
| Living with other people | -0.13 | 0.64 | 0.881 | 0.250 | 3.109 |  | -0.27 | 0.72 | 0.763 | 0.186 | 3.124 |  | 0.86 | 0.83 | 2.361 | 0.464 | 12.014 |  |
| Employment Status |  |  |  |  |  |  |  |  |  |  |  |  |  |  |  |  |  |  |
| Unemployed (ref) |  |  |  |  |  |  |  |  |  |  |  |  |  |  |  |  |  |  |
| Employed | -0.22 | 0.51 | 0.806 | 0.299 | 2.176 |  | -1.54 | 0.56 | 0.215 | 0.071 | 0.646 | ** | -0.06 | 0.55 | 0.943 | 0.322 | 2.758 |  |
| CIMD Neighborhood Deprivation |  |  |  |  |  |  |  |  |  |  |  |  |  |  |  |  |  |  |
| Quintile 1 (ref) |  |  |  |  |  |  |  |  |  |  |  |  |  |  |  |  |  |  |
| Quintile 2 | -0.51 | 0.89 | 0.602 | 0.105 | 3.453 |  | -1.82 | 0.79 | 0.162 | 0.034 | 0.769 | * | -0.01 | 0.90 | 0.989 | 0.169 | 5.800 |  |
| Quintile 3 | 0.50 | 0.93 | 1.642 | 0.266 | 10.141 |  | -0.93 | 0.85 | 0.394 | 0.074 | 2.100 |  | 0.77 | 0.94 | 2.150 | 0.338 | 13.668 |  |
| Quintile 4 | -0.32 | 1.12 | 0.729 | 0.080 | 6.604 |  | -1.24 | 1.05 | 0.290 | 0.037 | 2.275 |  | -0.41 | 1.22 | 0.665 | 0.061 | 7.242 |  |
| Quintile 5 | 0.34 | 0.94 | 1.407 | 0.221 | 8.956 |  | -1.16 | 0.86 | 0.315 | 0.058 | 1.700 |  | -0.12 | 1.02 | 0.891 | 0.121 | 6.578 |  |
| GAD-7 at Baseline | -0.05 | 0.07 | 0.947 | 0.819 | 1.095 |  | -0.14 | 0.08 | 0.872 | 0.751 | 1.013 | . | -0.23 | 0.08 | 0.792 | 0.683 | 0.918 | ** |
| PHQ-9 at Baseline | 0.09 | 0.07 | 1.092 | 0.961 | 1.241 |  | 0.25 | 0.07 | 1.290 | 1.123 | 1.481 | *** | 0.21 | 0.07 | 1.232 | 1.077 | 1.409 | ** |

Legend for p-value codes: 0.001 ‘***’ 0.01 ‘**’ 0.05 ‘*’ 0.1 ‘.’
