# Supplementary material for: Predicting CBT modality, treatment participation, and reliable improvements for individuals with anxiety and depression in a specialized mental health centre: a retrospective population-based cohort study
Source: BMC Psychiatry. 2024 May 23;24:390. doi: 10.1186/s12888-024-05817-w (PMC11112857; doi:10.1186/s12888-024-05817-w)
Supplement: Supplementary file 3 — Supplementary Material 3 [file 12888_2024_5817_MOESM3_ESM.docx]

Table S3. Regression results of a sensitivity analysis for showing post-treatment RCSI.

| Coefficients: | β | SE | Odds Ratio (OR) | 95% Confidence Interval (CI) for OR | |  |
| --- | --- | --- | --- | --- | --- | --- |
| Age | -0.04 | 0.02 | 0.962 | 0.933 | 0.991 | * |
| Sex |  |  |  |  |  |  |
| *Female (ref)* |  |  |  |  |  |  |
| Male | -0.06 | 0.43 | 0.943 | 0.405 | 2.221 |  |
| Living Status |  |  |  |  |  |  |
| *Living Alone (ref)* |  |  |  |  |  |  |
| Living with other people | 0.67 | 0.52 | 1.963 | 0.712 | 5.472 |  |
| Employment Status |  |  |  |  |  |  |
| *Unemployed (ref)* |  |  |  |  |  |  |
| Employed | 0.06 | 0.40 | 1.064 | 0.481 | 2.365 |  |
| CBT Modality type |  |  |  |  |  |  |
| *eCBT (ref)* |  |  |  |  |  |  |
| Group | 0.00 | 0.58 | 0.998 | 0.320 | 3.116 |  |
| Individual | -0.44 | 0.60 | 0.645 | 0.194 | 2.042 |  |
| Mixed | -0.53 | 0.59 | 0.591 | 0.183 | 1.881 |  |
| GAD-7 at Baseline | 0.09 | 0.05 | 1.094 | 0.992 | 1.210 | . |
| PHQ-9 at Baseline | 0.09 | 0.05 | 1.097 | 1.005 | 1.205 | * |
| CIMD Neighborhood Deprivation |  |  |  |  |  |  |
| *Quantile 1 (ref)* |  |  |  |  |  |  |
| Quantile 2 | -0.23 | 0.62 | 0.796 | 0.227 | 2.637 |  |
| Quantile 3 | -0.13 | 0.68 | 0.874 | 0.227 | 3.281 |  |
| Quantile 4 | 0.29 | 0.80 | 1.334 | 0.277 | 6.505 |  |
| Quantile 5 | -0.02 | 0.67 | 0.983 | 0.258 | 3.657 |  |

Legend for p-value codes: 0.001 ‘***’ 0.01 ‘**’ 0.05 ‘*’ 0.1 ‘.’
