# Supplementary material for: Predicting CBT modality, treatment participation, and reliable improvements for individuals with anxiety and depression in a specialized mental health centre: a retrospective population-based cohort study
Source: BMC Psychiatry. 2024 May 23;24:390. doi: 10.1186/s12888-024-05817-w (PMC11112857; doi:10.1186/s12888-024-05817-w)
Supplement: Supplementary file 4 — Supplementary Material 4 [file 12888_2024_5817_MOESM4_ESM.pdf]

Appendix

The sensitivity analysis was performed on a limited sample of our dataset (including all records from May 1, 2019, to January 1, 2020) to determine if the COVID-19 pandemic may have affected our results. Table S1 of the appendix provides each modality statistics based on our sample of the 155 clients who have participated in all four modalities before COVID-19 pandemic. There is no substantial difference in baseline characteristics between our original data and the sample. Still majority of our sample are females (70%) who are living with other people (84%) and are unemployed (57%). Over half of the sample have post-treatment RCSI and almost 80% of our sample completed six sessions and more. However, we cannot conclude that COVID-19 has any effect on our cohort. The regression results are also presented in appendix Tables S2 and S3, and they are like our original findings and do not corroborate that the COVID-19 pandemic may influence modality options and post-treatment RCSI. Age predicts the choice of group therapy versus eCBT, whereas employment status only explains the preference for eCBT over individual therapy. However, none of our socioeconomic characteristics are predictive of RCSI.
